# Supplementary material for: Functional Role of RING Ubiquitin E3 Ligase VdBre1 and VdHrd1 in the Pathogenicity and Penetration Structure Formation of Verticillium dahliae
Source: J Fungi (Basel). 2023 Oct 21;9(10):1037. doi: 10.3390/jof9101037 (PMC10608160; doi:10.3390/jof9101037)
Supplement: Supplementary file 1 [file jof-09-01037-s001.zip › jof-2544054-supplementary.pdf]

Supplementary files

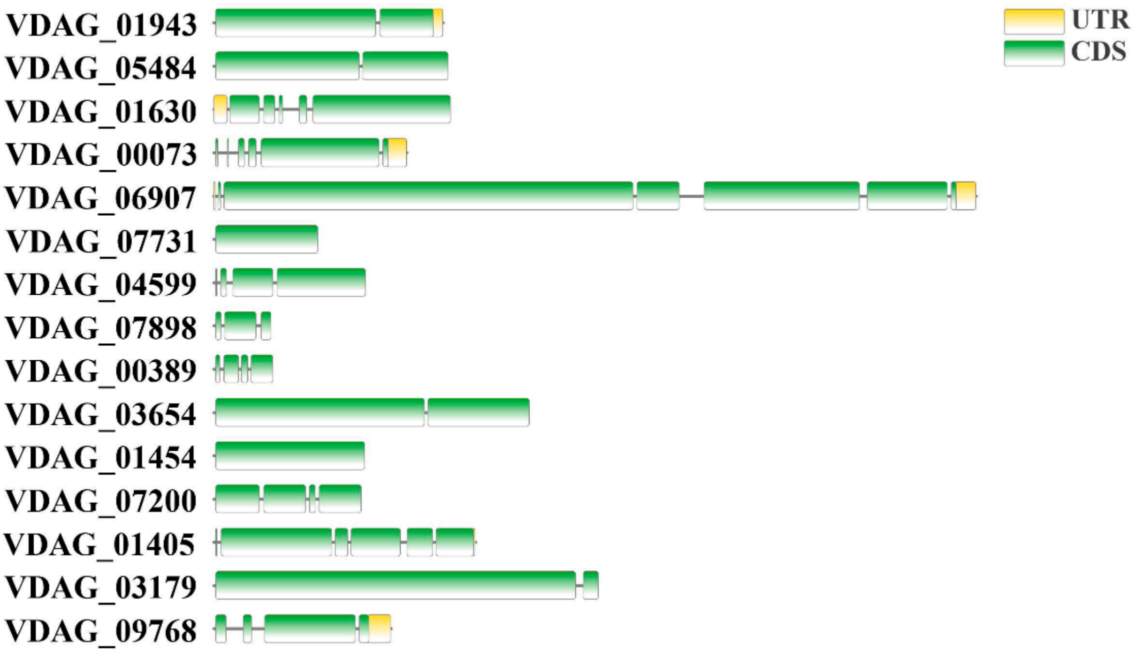

Fig. S1. Schematic for ubiquitin ligase E3 genes intron-exon structure in *Verticillium dahliae*. The black lines indicate introns, the green boxes represent exons. Genes are listed arranged in the order they appear on the phylogenetic tree and the number of exons per gene is listed on the right of gene symbol.

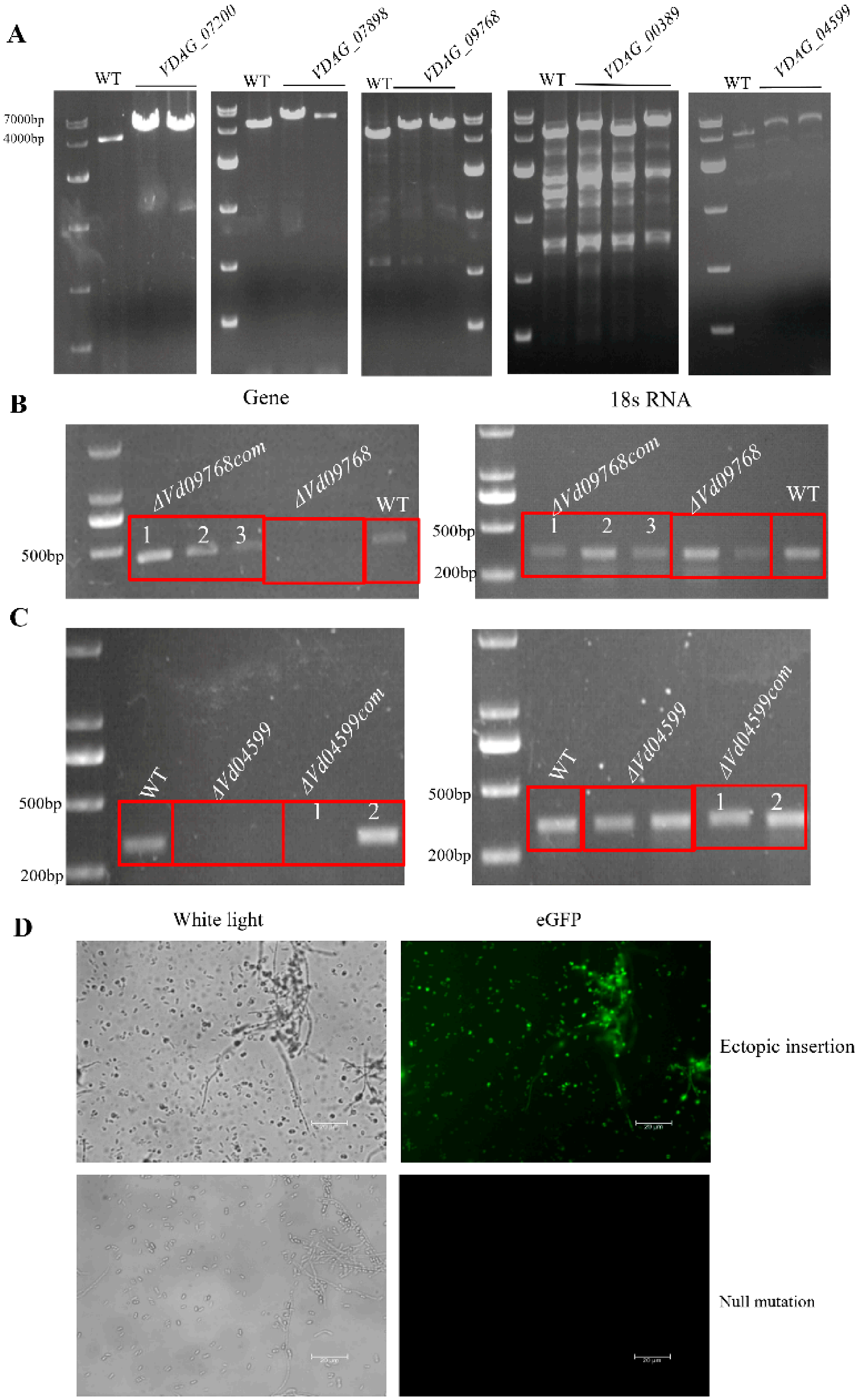

Fig. S2. Gene knockout and complementation in *Verticillium dahliae*. (A) The mutant was identified by gDNA amplification. (B-C) Verification of RNA level reveals the elimination of the gene in gene-deletion mutants and gene reintroduction in gene complementation strain. 18S RNA served as an internal control. (D) eGFP is not observed in null mutants but is present in ectopic insertion transformants or null mutants with ectopic insertions.

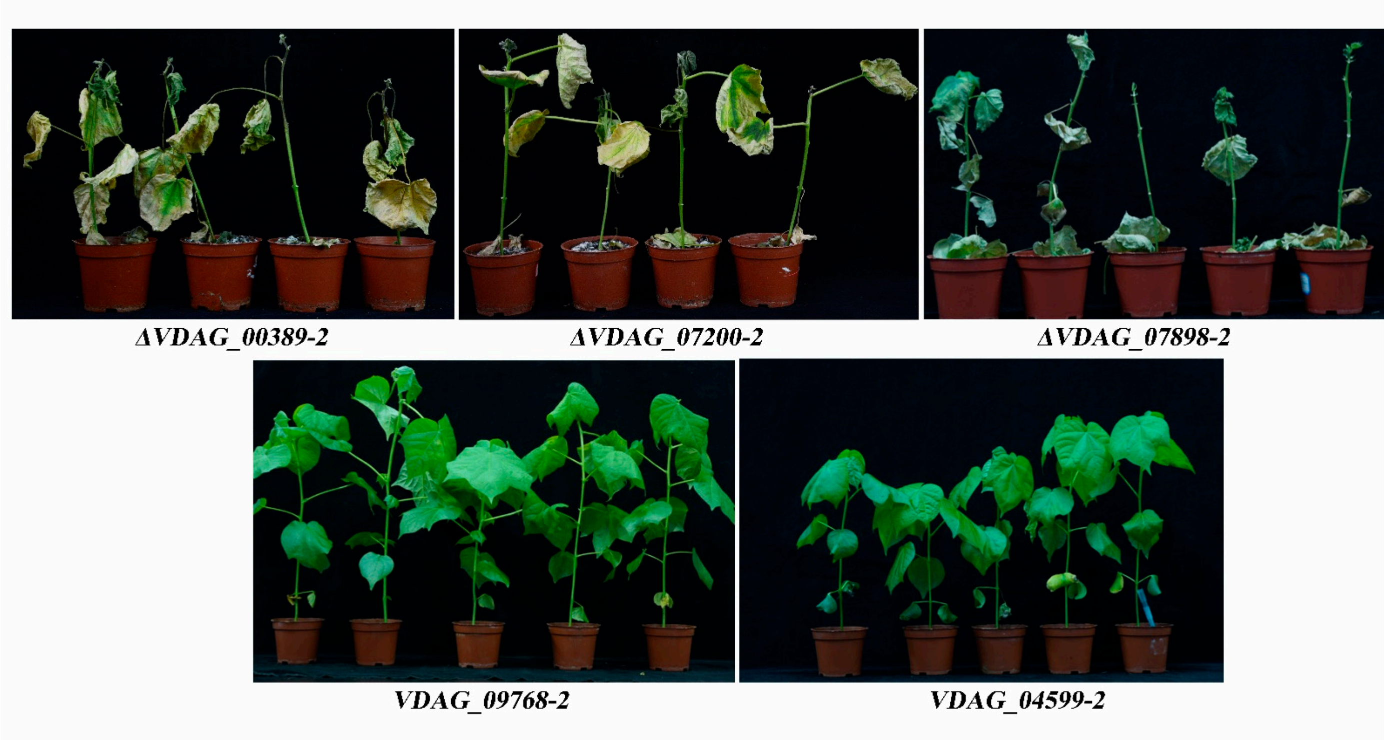

Fig. S3. Evaluation of pathogenicity on cotton plants. The *ΔVDAG_07200-2*, *ΔVDAG_07898-2*, and *ΔVDAG_00389-2* strains induced characteristic necrotic lesions on cotton plants, indicative of successful infection. In contrast, the *ΔVDAG_04599-2* and *ΔVDAG_09768-2* mutant strains were unable to elicit any symptoms associated with Verticillium wilt.

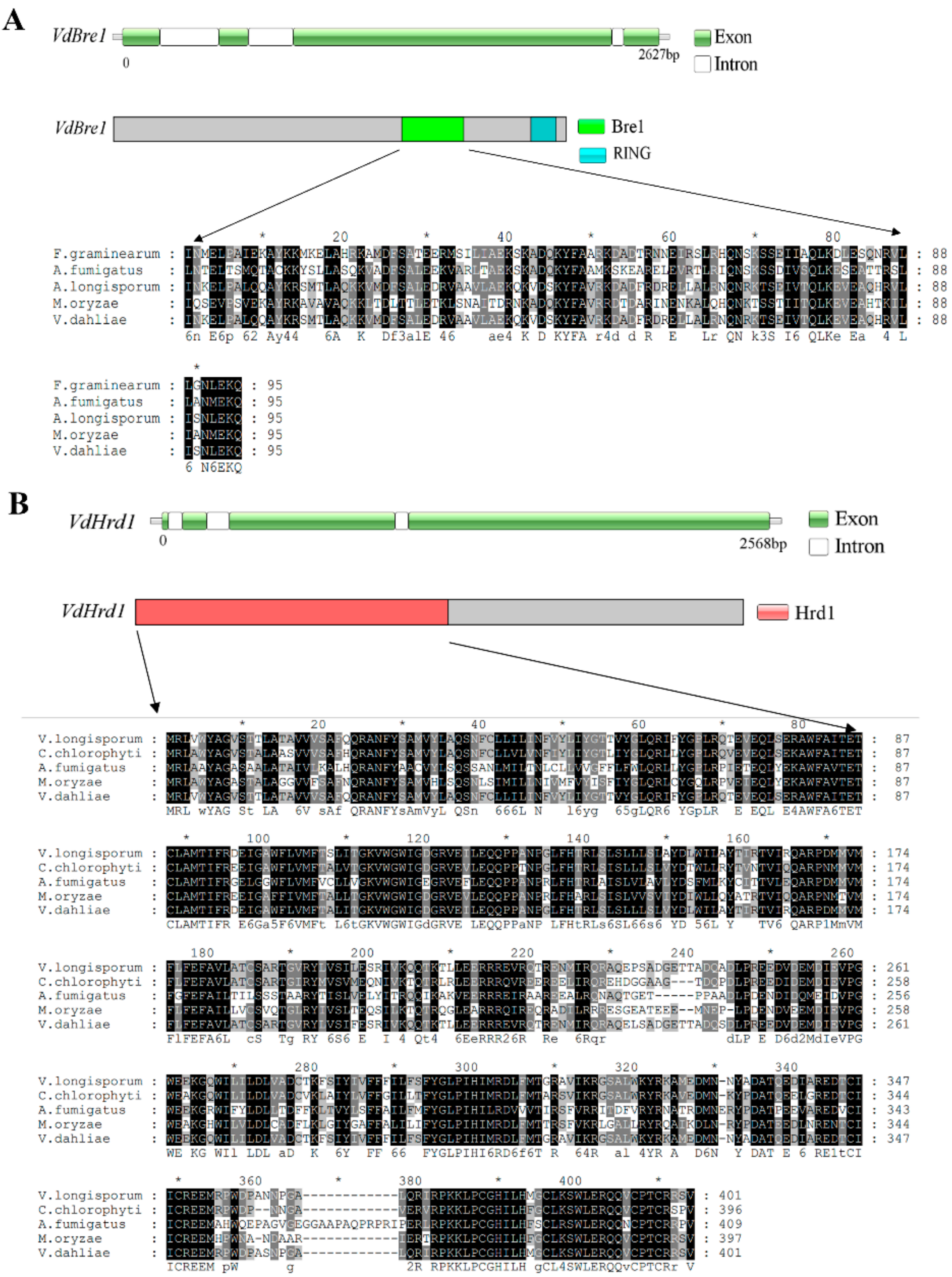

Fig. S4. Sequence analysis of *VdBre1* and *VdHrd1*. (A) Comparison between genomic and cDNA in *VdBre1*, and prediction of conserved domain. Multiple sequence alignment of Bre1 domain of *F. graminearum*, *A. fumigatus*, *V. longisporum, M. oryzae* and *V. dahliae*. (B) Comparison between genomic and cDNA in *VdHrd1*, and prediction of conserved domain. Multiple sequence alignment of Hrd1 domain of *V. longisporum*, *C. chlorophyte*, *A. fumigatus*, *M. oryzae* and *V. dahliae.* * represents the position every 10 amino acids.

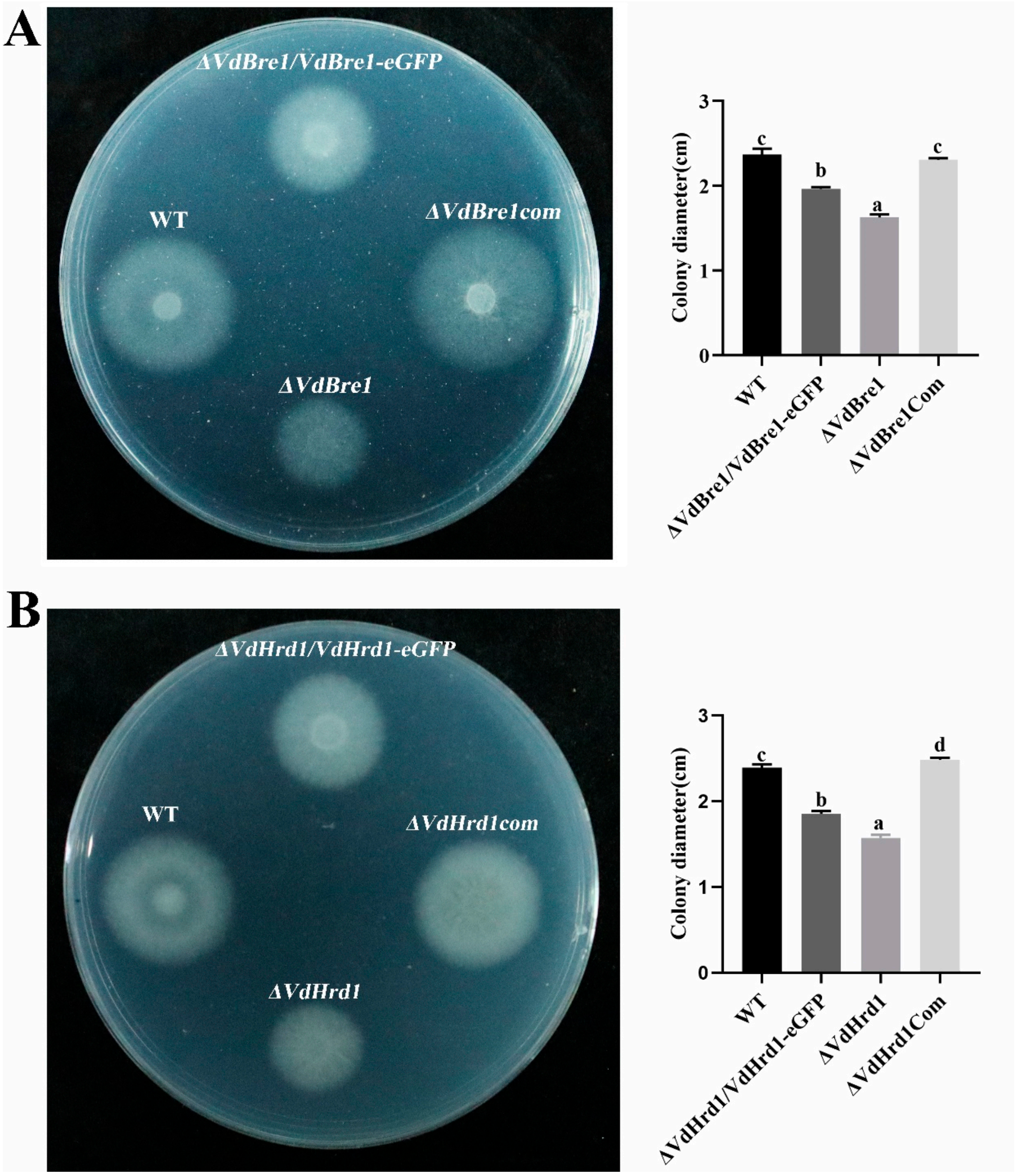

Fig. S5. Deletion of *VdBre1* and *VdHrd1* impairs hyphal growth of *Verticillium dahliae*. (A) Colony morphology of wild type (WT), *ΔVdBre1*, *ΔVdBre1com*, and *VdBre1/VdBre1-eGFP* strains after five days of growth on Czapek–Dox Medium. (B) Colony morphology of wild type (WT), *ΔVdHrd1*, *ΔVdHrd1com*, and *ΔVdHrd1/ΔVdHrd1-eGFP* strains after five days of growth on Czapek–Dox Medium. The diameter of colonies was measured and presented in a bar graph on the right side. The data are shown as the mean±standard error of the mean. In the figure caption, lowercase letters a, b, c, d are used to represent significant differences between different strains.

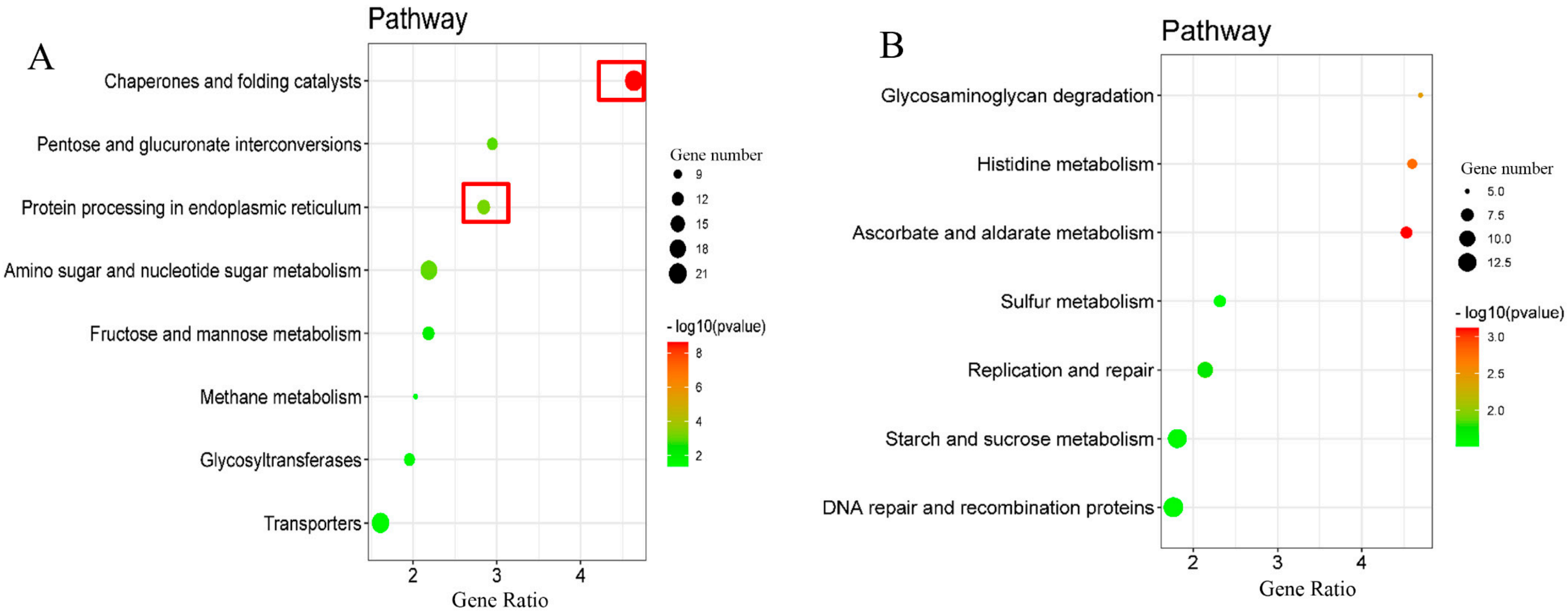

Fig. S6. KEGG enrichment analysis for differentially expressed genes between WT and *ΔVdHrd1*, screening the differentially expressed genes with absolute value of log2 ≥ 2 and P ≤0.01. (A) Genes with significantly up-regulated expression. (B) Genes with significantly down-regulated expression. The red box represents the enriched protein folding and processing-related genes.

Table S1 Primers used in this study.

Primer name	5’-3’	Use
VdBre1-5outF	ATCCACGGTTTGCTGGTCTG	Identify mutant
VdBre1-3outR	ACAGACAACGAATCCTTGCG	Identify mutant
VdHrd1-5outF	ATGGGACTGTTATACATCTG	Identify mutant
VdHrd-3outR	ACTCAGTACAGGTTTGATAG	Identify mutant
00389-5outF	TGTTTCTCCAATCTTTGCTG	Identify mutant
00389-3outR	ATCTCCCTCTCGTGATCAAG	Identify mutant
07200-5outF	TTGCACTTCGTAGCCGTCTC	Identify mutant
07200-3outR	ATCTCTGTCTGTAAAGTCTG	Identify mutant
07898-5outF	GCGTCGCATGGCACCAATCG	Identify mutant
07898-3outR	ACGAGACAGTCGACGCTCAG	Identify mutant
VdDer1-5outF	AGGATCCATCATATCACCTC	Identify mutant
VdDer1-3outR	ACTATACTGCTGACGATGAG	Identify mutant
VdBre1 -5F	AGAAGAGTAATCTAGATCGCTCTCGCCGCTTCCTG	Amplifying 5' homologous arms
VdBre1-5R	TTAGTGAGGGTTAATTGCGCGCGAATCATGGAAGGGTGAG	Amplifying 5' homologous arms
VdBre-3F	AGTTGTTCCCAGTGATCTTCGGACTCTTGAATGGTTCAAAG	Amplifying 3' homologous arms
VdBre1-3R	ATTACGAATTGGATCTGCGCATGCGGTTGCTGATG	Amplifying 3' homologous arms
VdHrd1-5F	AGAAGAGTAATCTAGCCTGGTGCTGAAGCATCAAG	Amplifying 5' homologous arms
VdHrd1-5R	TTAGTGAGGGTTAATTGCGCGGAATTGGTCGCGCTGCGTG	Amplifying 5' homologous arms
VdHrd1-3F	AGTTGTTCCCAGTGATCTTCGGACTGACCATGGACAGATTG	Amplifying 3' homologous arms
VdHrd1-3R	ATTACGAATTGGATCTCAGTCATATGCCTGCCCAC	Amplifying 3' homologous arms
00389-5F	AGAAGAGTAATCTAGAGCGGTGGTGAAACGACTTG	Amplifying 5' homologous arms
00389-5R	TTAGTGAGGGTTAATTGCGCATCGTGTCGATTGGGCTGTG	Amplifying 5' homologous arms
00389-3F	AGTTGTTCCCAGTGATCTTCGCTACAGTTACAAGTATCAAG	Amplifying 3' homologous arms

00389-3R	ATTACGAATTGGATCATTTGCTCATCTCTTCGATG	Amplifying 3' homologous arms
07200-5F	AGAAGAGTAATCTAGAGATGTCGAGGCCAATAGAG	Amplifying 5' homologous arms
07200-5R	TTAGTGAGGGTTAATTGCGCTCAAATGCCAGCCAGACTCG	Amplifying 5' homologous arms
07200-3F	AGTTGTTCCCAGTGATCTTCGTGATCACTTGCGTATCCTAG	Amplifying 3' homologous arms
07200-3R	ATTACGAATTGGATCAAGTCGCCGTAAAGACTAAG	Amplifying 3' homologous arms
07898-5F	AGAAGAGTAATCTAGGTAATTGATAGGCTCTGTTG	Amplifying 5' homologous arms
07898-5R	TTAGTGAGGGTTAATTGCGCGAATGTGTTGCTCGTTGCAG	Amplifying 5' homologous arms
07898-3F	AGTTGTTCCCAGTGATCTTCGGCATATCTAGTCTGTTCAGC	Amplifying 3' homologous arms
07898-3R	ATTACGAATTGGATCTGATGTGGAAGCCGTATTCG	Amplifying 3' homologous arms
VdDer1-5F	ACGACAAATAAGAATTCTCGTTTGGCATGTATCGCAC	Amplifying 5' homologous arms
VdDer1-5R	ATTGCATGCTCTCACGTCGAAGCGGAAGTCTTCCACCATC	Amplifying 5' homologous arms
VdDer1-3F	CTGTGGCGTTGGCACGTCGATGGACGGCACAAACAACGAC	Amplifying 3' homologous arms
VdDer1-3R	TCGAGGGGGGGCCCGGTACCTCCACTTGGGTTCGTCTTTG	Amplifying 3' homologous arms
Chle-F	GATGGTGGAAGACTTCCGCTTCGACGTGAGAGCATGCAAT	Amplifying chlorosulfuron resistance gene
Chle-R	GTCGTTGTTTGTGCCGTCCATCGACGTGCCAACGCCACAG	Amplifying chlorosulfuron resistance gene
TubllinF	ACGGTCGCTACCTGACCTGC	Identify mutant
TubllinR	CATCTCGTCCATACCCTCACCA	Identify mutant
VdHrd1-BDF	TGCCTTGCGATGACCATCTT	Identify mutant
VdHrd1-BDR	GACCAGATATCGGACGCCAG	Identify mutant
VdBre1-BDF	CCTCCCAGCAAGCGTCAT	Identify mutant
VdBre1-BDR	ATTCGGCGTGATTTGTCC	Identify mutant
VdDer1-BDF	GGTCTCGACGTAATCCCGAC	Identify mutant
VdDer1-BDR	TGACCACGCCCATAATCTCG	Identify mutant
VdHrd1-comF	CGGCCAGTGCCAAGCTTAGCATCAAGCTTCTGCATGC	Amplifying complement
VdHrd1-comR	CAGTTAACGTCGAATTCAAGTGCTCATGACGCACAGC	Amplifying complement
VdBre1-comF	CGGCAAGTGCCAAGCTTTGTTTCCTCTTCCGAGGCTG	Amplifying complement

VdBre1-comR	CAGTTAACGTCGAATTCGCTTGCTGTTCATGGTGCTC	Amplifying complement
Bre1-GFPF	CAGGGAGTGGTTCCGGCAGCATGCCTGTAGCCATCAAGCC	Subcellular localization
Bre1R	TCATCTTCTGGAATTCCTAGTGGTGAACAGACAACGAATCC	Subcellular localization
GFP-Bre1R	GGCTTGATGGCTACAGGCATGCTGCCGGAACCACTCCCTG	Subcellular localization
eGFPF	TCTAGAGGATCCTTAATTAAATGGTGAGCAAGGGCGAGG	Subcellular localization
eGFPR	TCATCTTCTGGAATTCTTACTTGTACAGCTCGTCCATGCC	Subcellular localization
GFP-Hrd1R	GCATACCACACGAGCCGCATGCTGCCGGAACCACTCCCtG	Subcellular localization
Hrd1-GFPF	CAGGGAGTGGTTCCGGCAGCATGCGGCTCGTGTGGTATGC	Subcellular localization
Hrd1R	TCATCTTCTGGAATTCCTACGAGGCGTCGCCCTCC	Subcellular localization
Sep5F	CAATCTTCAAATCTAGAATGTCGTCTTCAGCTACTTT	Septin localization
Sep5R	CCACCGCCTCCACTAGTTTGCTGTCATTCTCGCCGT	Septin localization
G418eGFPF	TCTAGAGGATCCTTAATTAAATGGTGAGCAAGGGCGAGG	Septin localization
G418eGFPR	AGTTAACGTCGAATTCCTACTTGTACAGCTCGTCCATGCC	Septin localization
07507F	GTTCAAGACGGTCGGCAT	qPCR
07507R	TTATTTGCTCGTGTCAATGAC	qPCR
09868 F	CTGCACCGACACGTACAC	qPCR
09868R	TCTGCTTGTGCTCGTACT	qPCR
02241 F	ACTGGCAATATGGTCTCA	qPCR
02241R	AATCCACTTGTCATCCTTATC	qPCR
07771 F	CAGGTCGTCGGCAGTAAC	qPCR
07771R	GAAGGCGTTGAGGAACCA	qPCR
05865 F	GTTCCCAAGATTGTCCTT	qPCR
05865R	CAGACAGCGGATATCTAC	qPCR
07080 F	ATGTTCAAGCGGAGTCAA	qPCR
07080R	CACCACTAGCATGTCCATA	qPCR
00583 F	ACAGACGGTAATAATCAT	qPCR

00583R	ATTCACATACTCATCAGA	qPCR
07881 F	ACGCTGTACGTCAACAAG	qPCR
07881R	TTGCCAAAGATGAAGTCG	qPCR
07659F	CTCACAATGTCTCGTCAA	qPCR
07659R	ATCTGGTCTTCTACTCTCA	qPCR

Table S2 Function of differentially expressed genes in this study.

**Gene ID**	**GenBank accession number**	**Function**
VDAG_07507	XP_009654707.1	Alcohol dehydrogenase
VDAG_02241	XP_009656565.1	Linoleate diol synthase
VDAG_07771	XP_009650832.1	Oxidoreductase
VDAG_05865	XP_009653557.1	Methylitaconate delta2-delta3-isomerase
VDAG_07881	XP_009650942.1	Pectinesterase
VDAG_07695	XP_009654895.1	chorismate synthase
VDAG_02709	XP_009651657.1	Pectate lyase
VDAG_05344	XP_009650534.1	Pectate lyase B
VDAG_06165	XP_009657474.1	Endo-1,4-beta-xylanase A
VDAG_07200	XP_009654400.1	SCF E3 ubiquitin ligase complex F-box protein grrA
VDAG_07898	XP_009650959.1	E3 ubiquitin-protein ligase CHIP
VDAG_00389	XP_009650061.1	E3 ubiquitin-protein ligase CCNB1IP1

Table S3 *Verticillium dahliae* strains used in this study.

Strain	Genotype description	Reference
V991	Wild type	Xie et al., 2017
ΔVdBre1	VdBre1 deletion mutant	This study
ΔVdHrd1	VdHrd1 deletion mutant	This study
ΔVDAG_00389	Vd00389 deletion mutant	This study
ΔVDAG_07200	Vd07200 deletion mutant	This study
ΔVDAG_07898	Vd07898 deletion mutant	This study
ΔVdBre1com	VdBre1 complementary strain	This study
ΔVdHrd1com	VdHrd1 complementary strain	This study
WT/*eGFP*	Transformant of v991 expressing *eGFP*	This study
ΔVdBre1/eGFP	Transformant of *ΔVdBre1* expressing *eGFP*	This study
ΔVdHrd1/eGFP	Transformant of *ΔVdHrd1* expressing *eGFP*	This study
WT/*VdSep5-eGFP*	Transformant of v991 expressing *VdSep5-eGFP*	This study
ΔVdBre1/VdSep5-eGFP	Transformant of *ΔVdBre1* expressing *VdSep5-eGFP*	This study
ΔVdHrd1/VdSep5-eGFP	Transformant of *ΔVdHrd1* expressing *VdSep5-eGFP*	This study
WT/*VdBre1-eGFP*	Transformant of v991 expressing *VdBre1-eGFP*	This study
WT/*VdHrd1-eGFP*	Transformant of v991 expressing *VdHrd1-eGFP*	This study
WT/ *VdBre1-eGFP /mCherry-NLS*	Transformant of WT/*VdBre1-eGFP expressing mCherry-NLS*	This study
VdBre1/VdBre1-eGFP	Transformant of *VdBre1 expressing VdBre1-eGFP*	This study
VdHrd1/VdHrd1-eGFP	Transformant of *VdHrd1* expressing *VdHrd1-eGFP*	This study
